# Efficacy of propolis-based mouthwashes on dental plaque and gingival inflammation: a systematic review

**DOI:** 10.1186/s12903-020-01185-5

**Published:** 2020-07-10

**Authors:** Esam Halboub, Sadeq A. Al-Maweri, Mohammed Al-Wesabi, Ahlam Al-Kamel, Anas Shamala, Amani Al-Sharani, Pradeep Koppolu

**Affiliations:** 1 Department of Maxillofacial Surgery and Diagnostic Sciences, College of Dentistry, Jazan University, Jizan, Kingdom of Saudi Arabia; 2 Department of Oral Medicine, Oral Pathology and Oral Radiology, Faculty of Dentistry, Sana’a University, Sana’a, Yemen; 3 Department of Oral Medicine and Diagnostic Sciences, AlFarabi Colleges of Dentistry and Nursing, Riyadh, Saudi Arabia; 4 Department of Preventive and Biomedical Science, Faculty of Dentistry, University of Science and Technology, Sana’a, Yemen; 5 Department of Preventive Dental Sciences, College of Dentistry, Dar Al Uloom University, Riyadh, Saudi Arabia

**Keywords:** Propolis mouthwash, Chlorhexidine, Efficacy, Plaque, Gingivitis

## Abstract

**Background:**

This systematic review of randomized clinical trials aimed to evaluate the available evidence regarding the efficacy of propolis-based mouthwash on dental plaque and gingival inflammation.

**Methods:**

PubMed, Scopus, and Web of Science databases were searched up to November 2019. Clinical trials that evaluated the efficacy of propolis mouthwashes compared with chlorhexidine (CHX) were included. The primary outcomes comprised dental plaque and/or gingival inflammation. Two authors assessed the risk of bias using the Cochrane tool. Due to marked heterogeneity of the available data, studies were assessed qualitatively, and no metaanalysis was performed.

**Results:**

Nine clinical trials, comprising 333 subjects, fulfilled the eligibility criteria. Most of the included studies showed high risk of bias. Overall, propolis mouthwashes showed good efficacy on plaque and gingivitis in all of the included studies. Out of the eight studies that reported on plaque index, 5 studies found equal efficacy of propolis and CHX in reducing plaque, two studies found superior efficacy in favor of CHX, while one study found superior efficacy in favor of propolis. Six studies assessed gingival inflammation outcome, four of which reported better results with propolis, while two studies reported comparable results.

**Conclusions:**

The results suggest that propolis-based mouthwashes have potential benefits in reducing plaque and gingival inflammation. However, methodological limitations along with small sample sizes in some of the included studies weaken the strength of the evidence. Therefore, further well-designed clinical trials with large sample sizes and adequate follow-up period are recommended to discern the efficacy of propolis mouthwash on plaque and gingivitis.

## Background

Dental plaque is the main etiological factor of periodontal diseases, mainly plaque-induced gingivitis [1, 2]. The latter is the most common inflammatory condition of the gingiva, affecting a large segment of the population [1]; hence, gingivitis is frequently encountered in the daily dental practice [3]. Gingivitis is a preventable and reversible condition. However, if left untreated, gingivitis can progress into periodontitis and eventually tooth loss and edentulism [3]. Thus, dental plaque must be brought under control via proper oral hygiene.

Improper oral hygiene is the main risk factor for periodontal diseases, irrespective of the age [1, 2]. Therefore, adequate and proper plaque control measures have to be applied regularly [2]. Nevertheless, mechanical methods might not be feasible and/or sufficient [4]. Hence, chemical preparations such as antimicrobial mouthwashes have been suggested either as an adjunctive or as a replacement for mechanical plaque control [4].

Owing to their ease of use and availability over the counter, the public highly appreciate mouthwashes. Chlorhexidine mouthwash (CHX) is considered as the gold standard antiplaque agent [4]. CHX (a broad spectrum cationic, bisbiguanide antiseptic) is used in diverse medical fields, mainly due to its antibacterial nature [4]. CHX seems to exert an instant bactericidal effect followed by an extended bacteriostatic effect [4]. Nevertheless, long term-use of CHX has been linked with numerous adverse effects including altered taste perception, staining of teeth and tongue, burning sensation and genotoxicity of buccal epithelial cells [5, 6]. Consequently, pharmaceutical companies have long been attempting to formulate natural-derived oral care products [7–9]. Among these, propolis -a natural resinous material produced by honey bee- has recently been proposed as an alternative anti-plaque mouthwash [8, 10]. The chemical composition of propolis includes 50% resin, 30% wax, 10% aromatic and essential oils, 5% pollen and 5% other constituents [10–12]. The flavonoids are the main biologically dynamic constituents of propolis extracts, suggesting homogeneity of propolis preparations, making their use harmless compared to many other synthetic products [13]. Propolis has shown strong antimicrobial and anti-inflammatory properties, making it a good candidate for treatment and prevention of oral diseases [12, 14–16]. In this context, a number of clinical trials have investigated the efficacy of propolis on plaque and gingivitis, and conflicting results were reported [8, 14, 17, 18]. While some studies found equal or even superior efficacy in favor of propolis [17–22], others reported better efficacy with CHX [8, 14].

Owing to the contradictory reports, the current systematic review aimed to evaluate the available evidence on the efficacy of propolis-based mouthwashes in comparison with CHX on dental plaque and gingival inflammation.

## Methods

### Focused question

The present systematic review strictly adhered to the Preferred Reporting Items for Systematic Reviews and Meta-Analyses (PRISMA) guidelines and PICO principles [23]. The protocol of this systematic review was also registered at PROSPERO (ID = CRD42020156187). The focused PICO question was: compared to CHX, what is the efficacy of propolis-based mouthwashes on plaque and gingivitis?

### Eligibility criteria

The following eligibility criteria were applied: 1) Study design: Randomized controlled trials (RCT); 2) Participants: Systemically healthy adults aged ≥18 years; 3) Intervention: propolis-based mouthwash of any concentration; 4) Comparison group: CHX mouthwash of any concentration; 5) Outcomes: gingival inflammation and/or plaque index; 6) Follow-up period: any time with no restriction on the follow-up time; and 7) Language: articles published in English.

### Literature search

A comprehensive literature search of PubMed/Medline, Scopus, and Web of Science (ISI) databases was conducted in December 2019 for all relevant studies from the date of inception up to and including November 2019. A combination of the following keywords and Medical Subject Heading (MeSH) terms were used: (“propolis” OR “honey” OR “honeybee” OR “bee glue”) AND (“gingivitis” OR “gingival health” OR “gingival disease” OR “dental plaque” OR “dental health” OR “periodontitis” OR “periodontal health” OR “periodontal disease”). The titles and abstracts of the retrieved studies were screened independently by two reviewing groups composed of two reviewers each (AS, AM) and (AA, AA), and irrelevant studies were excluded. Disagreements, if any, were resolved by discussion or by a reviewer not involved in the screening. Full-text of the potentially eligible studies were obtained and evaluated independently by the two reviewing groups for inclusion. Respective authors were contacted whenever deemed necessary. Additionally, the references of the retrieved studies were hand-searched for any additional studies. One of the included studies lacked some important data, and hence the respective authors were contacted via email; thankfully, the authors responded and provided all missing information.

### Risk of bias assessment (quality assessment)

Risk of bias of the included studies was carried out independently by two reviewers (AS and AA) according to the Cochrane risk of bias assessment tool [24]. The following domains were assessed: sequence generating; allocation concealment; blinding of participants and personnel; blinding of outcome assessment; incomplete outcome data; selective outcome reporting; and other sources of bias. Accordingly, the overall risk of bias of each included study was judged as either: low, all criteria were of low risk; unclear, at least one criterion was evaluated to be of unclear risk but no criterion of high risk; or high, at least one criterion with high risk of bias [24].

### Data extraction

The following data were extracted and tabulated by two authors (AS and AM): authors and year of the study, the study design, country, number of participants, mean ages, gender, concentration of propolis and CHX, frequency of use per day, follow-up period, side effects, clinical parameters; and main outcomes.

## Results

### Study selection

A total of 849 articles were retrieved, 248 of which were duplicates and thus were removed. The titles and abstracts of the remaining 601 articles were screened independently by the two-reviewing groups, and 559 were excluded because they did not fulfill the eligibility criteria. The full-text of the remaining 42 articles were obtained and evaluated for inclusion. Of these, 33 articles were excluded for various reasons (list of the excluded studies and the reason of exclusion are presented in supplementary Table 1). Eventually, 9 studies fulfilled the eligibility criteria and were processed for data extraction.

### General characteristics of the included studies

General characteristics of the included studies are presented in Table 1. Nine randomized clinical trials (RCT) involving 333 patients were included in this systematic review [8, 11, 14, 17–22] (Table 1). Four studies were conducted in India [14, 20–22], two in Brazil [17, 18], one in Iran [11], one in Iraq [19], and one in the UK [8]. Number of subjects ranged between 10 and 60 subjects. All included studies enrolled healthy subjects with no history of systemic diseases. Medications or dentifrices known to affect the intervention were considered as exclusion criteria in all included studies. Three studies [11, 17, 21] reported excluding smokers, while the remaining studies did not provide any information about smoking status of the participants. The evaluation period ranged from 5 to 28 days. Six studies [11, 14, 17, 19, 20, 22] reported the age of the participants (age range: 15–70 years). Only three studies [11, 19, 20] reported the gender of the participants; the number of female subjects varied among the studies and ranged from 2 to 27. Two studies [8, 21] neither mentioned the age nor the gender of the subjects (Table 1).

**Table 1:** General characteristics of the included studies

Authors/Year (country)	Type of study	M/F (Age range)	Study Groups (Sample size)	Index	Diagnosis of gingivitis	Concentration / Composition of propolis	Professional procedure/ Hygiene during trial	Smoking/ Ortho. appliance	Frequency of mouthwashes used/ durations of rinsing per time	Follow-up Period	Study outcome
Murray et al. 1997 [8] (UK)	Double blind three group parallel study	41	G1:propolis (*n* = 13) G2:CHX 0.12% (*n* = 14)	PI	–	Propolis Extract 10% in ethanol, polyoxyethelers, Hydrogenated castor oil, sodium nipasept and water	Scaling and polishing, 1 week before.	NM	Twice a day for 1 min	5 days	CHX was significantly more effective than propolis in reducing plaque.
Santiago 2018 [18] (Brazil)	Three group parallel study	(20–40 years)	G1:propolis (n=) G2:CHX (n=) G3:propolis plus CHX (n=)	PI (PHP)	–	Propolis-containing mouthwash	Dental plaque was removed at the beginning of the stuy, standard toothbrushes, toothpaste and floss	NM	NM	14 days	Propolis and CHX showed comparable results in reducing plaque
Porwal et al. 2018 [22] (India)	Three group parallel Randomized Clinical trial	(20–40)	G1: propolis (*n* = 10) G2: CHX 0.2% (*n* = 10)	PI & GI	NM	Raw propolis diluted with distilled water (1:1)	Thorough scaling and root planning	Ortho. treatment or bridge work were excluded	10 ml mouthwash twice daily	28 day	Propolis was comparable to CHX in reducing plaque but more effective in reducing gingival inflammation.
Krishna et al. 2019 [20] (India)	Randomized Controlled clinical trial	27/18 (18–70)	G1: propolis (*n* = 15) G2: CHX 0.2% (*n* = 15)	PI & GI	Chronic generalized gingivitis	5% propolis	Scaling and root planning	NM	10 ml daily Twice at morning & twice at night	6 weeks	Propolis showed significantly better efficacy than CHX in reducing GI and PI .
Dodwad and Kukreja 2011 [14] (India)	A single blind three-group parallel study	(18–50)	G1: propolis (*n* = 10) G2: CHX 0.2% (*n* = 10)	PI & GI	Chronic generalized gingivitis	NM	Professionally scaled and polished, and to refrain from all other oral hygiene measures until the final examination	NM	Twice a day for one min.	5 days	CHX showed slight better efficacy than propolis in reducing plaque. Propolis was marginally better in reducing gingival inflammation than
Savita et al. 2018 [21] (India)	Randomized Clinical Crossover Study	NM	G1: Propolis (*n* = 20) G2: CHX 0.2% (*n* = 20)	PI & GI	Moderate to severe gingivitis	20% Propolis Food grade alcohol, Glycerine, Sorbitol, Menthol, Saccharin, Benzyl alcohol and flavor Anise Mint	Supragingival ultrasonic scaling was carried out in the same appointments	Smoking, tobacco chewing.	10 ml twice daily for two Weeks	2 weeks	Propolis and CHX were equally effective in reducing PI and GI.
Abdullah et al. 2003 [19] (Iraq)	Double-blind crossover design	10 8/2 (20–24)	G1: propolis (*n* = 10) G2: propolis (ethanol extract) (*n* = 10) G3: CHX 0.2% (*n* = 10)	PI	–	(0.5, 1% water extract of propolis, 0.5, 1% ethanolic extract of propolis) with distilled water	Professional scaling & polishing, daily tooth brushing & flossing then abstain all mechanical tooth cleaning effort for the next five days	No any appliance	15 ml three times daily for one minute each time	5 days	Propolis 1% ethanolic extract was as effective as CHX in reducing plaque
Dehghani et al. 2019 [11] (Iran)	Triple-blind parallel-group Clinical trial.	10\27 (15–35)	G1:popolis (*n* = 18) G2: CHX 0.12% (*n* = 19)	PI &GI	Mild to moderate gingivitis	Propolis aqueous extract, Propolis was 1% with a salt concentration of 0.25%, oil of saffron and flavor	Subjects continue their usual daily brushing method (tooth brush and dental floss) during the study period	Non- smokers under fixed ortho. Treatment	15 ml, twice a day after brushing, for 1 min	3 weeks	Both mouthwashes were equally efficacious in reducing plaque and gingivitis
Anauate-Netto et al. 2014 [17] (Brazil)	Randomized double-blind, placebo-controlled clinical trial	(18–55 yeas)	G1: propolis (*n* = 20) G2: CHX 0.12% (*n* = 20)	PBS	No clinical signs of periodontal disease	Alcohol-free, 2% typified propolis, mint Flavor, water, polioxyethelers, sorbitol and blue color	None	Not being a current smoker	15 ml of mouthrinse twice a day after ordinary oral hygiene procedures	28 days	Propolis mouthrinse was superior to CHX in reducing mean PBS (*P* < 0.05).

*GI* Gingival Index, *PI* Plaque Index, *CHX* Chlorhexidine, *NM* Not mentioned, *PHP* Patient Hygiene Performance, *PBS* Papillary Bleeding Score

### Intervention and comparison groups

All included studies compared propolis-based mouthwashes with CHX [8, 11, 14, 17–22] (Table 1). Eight studies [8, 11, 14, 17, 18, 20–22] used the raw propolis, while one study [19] used two formulations of propolis namely, water extract poroplis and ethanol extract propolis (Table 1). With regards to the control group, seven studies reported on CHX concentrations: five studies used CHX 0.2% [14, 19–22], and two studies used CHX 0.12% [8, 17] (Table 1).

### Outcome measures

Dental plaque was assessed in eight included studies [8, 11, 14, 18–22]. Methods of recording the plaque were highly variable across the included studies: three studies used Silness and Loe plaque index (PI) [8, 11, 19]; three used Turesky-Gilmore-Glickman modification of Quigley-Hein PI [14, 21, 22]; and one used the Patient Hygiene Performance [18]. One study did not mention the type of plaque scoring index [20]. With regards to gingival inflammation, six studies reported on this outcome [11, 14, 17, 20–22]. Three of these studies [11, 14, 21] used Loe and Silness gingival index (GI), and one study [22] used the modified GI by Lobene. Contrastingly, one study used the papillary bleeding index [17]; whereas one study did not provide any information about the scoring index [20].

### Main outcomes

Out of the eight studies [8, 11, 14, 18–22] that reported on PI, 5 studies [11, 18, 19, 21, 22] found comparable results between the two groups and one study [20] found propolis-based mouthwashes superior to CHX in reducing plaque. Conversely, two studies reported CHX superior to propolis-based mouthwashes in reducing dental plaque [8, 14] (Table 1 & supplementary Table 2).

Out of the six studies that reported on gingival inflammation outcome, four studies [14, 17, 20, 22] reported propolis-based mouthwashes to be superior to CHX in reducing gingival inflammation. Two studies [11, 21] found comparable results in reducing gingival inflammation (Table 1 and supplementary Table 2).

### Side effects

Eight studies [8, 11, 14, 18–22] reported no side effects of the assessed mouthwashes. One study [17] reported some adverse effects (e.g., burning, taste alterations, yellow teeth, breath alteration, tongue burning, bitter taste) in 23 participants of CHX group, and in 7 participants of propolis group.

### Risk of bias of the included studies

The risk of bias assessment results are summarized in Table 2. Only two studies [11, 17] were at low risk of bias, while the majority of included studies [8, 14, 18–20, 22] were at high risk of bias and one study [21] showed unclear risk of bias (Table 2).

**Table 2:** A summary of the risk of bias assessment of the included studies

Study	Sequence generation	Allocation concealment	Blinding of participants	Blinding of outcome	Incomplete outcome data	Selective outcome reporting	Other potential threats to validity	Estimated risk of bias within study
Anauate-Netto et al.	Low risk	Low risk	Low risk	Low risk	Low risk	Low risk	Low risk	Low risk
Dehghani et al.	Low risk	Low risk	Low risk	Low risk	Low risk	Low risk	Low risk	Low risk
Abdullah et al.	Low risk	High risk	Low risk	High risk	Low risk	Low risk	Low risk	High risk
Dodwad and Kukreja	Low risk	Low risk	Low risk	High risk	Low risk	Low risk	Low risk	High risk
Savita et al.	Low risk	Low risk	Unclear risk	Unclear risk	Unclear risk	Low risk	Low risk	Unclear risk
Santiago et al.	High risk	High risk	High risk	High risk	Unclear risk	Low risk	High risk	High risk
Murray et al.	Low risk	Low risk	Low risk	Unclear risk	Low risk	Low risk	Low risk	High risk
Porwal et al.	Low risk	Low risk	Unclear risk	High risk	Low risk	Low risk	Low risk	High risk
Krishna et al.	Low risk	Low risk	Unclear risk	High risk	Low risk	Low risk	Low risk	High risk

## Discussion

To the best of our knowledge, this is the first systematic review that assessed the available evidence regarding the efficacy of propolis-based mouthwashe in comparison to CHX in reducing plaque accumulation and gingival inflammation. Overall, the findings of the qualitative analysis suggest that propolis-based mouthwashes are comparable to CHX in reducing plaque, but more efficacious in reducing gingival inflammation. Additionally, the results showed that propolis-based mouthwashes are safe with no or minimal reported side effects. Nevertheless, these findings should be interpreted with caution, given some methodological weaknesses discussed in the subsequent sections.

One of the main outcomes assessed in the present review was the efficacy of propolis-based mouthwashea in reducing dental plaque. The results showed positive antiplaque efficacy of propolis comparable to CHX, the gold standard mouthwash for plaque. Propolis has been shown to have antibacterial properties against different strains of aerobic and anerobic oral pathogens, probably due to the flavonoid content of propolis [12, 15, 18]. Another important finding of the present systematic review is the potent anti-gingivitis effects of propolis-based mouthwashes. Among the included nine studies, six randomized clinical trials [11, 14, 17, 20–22] included gingival inflammation as an outcome; of these, four studies [14, 17, 20, 22] reported better outcomes with propolis-based mouthwashes, whereas two studies [11, 21] reported propolis-based mouthwashes as efficacious as CHX in reducing gingival inflammation. The superior efficacy of propolis-based mouthwashes in reducing gingival inflammation can be attributed to the propolis’ potent anti-inflammatory properties. Recent in-vivo and in-vitro studies have shown that propolis inhibits prostaglandin production through inhibiting lipoxygenase and cyclooxygenase enzymes, resulting in a rapid and potent reduction in pain and tissue inflammation [12, 25].

Undoubtedly, CHX is the most widely used mouthwash for plaque and gingivitis [5]. However, CHX has been linked with numerous side effects including, but not limited to, taste changes, and teeth staining. The present systematic review revealed that propolis-based mouthwashes were safe and well-tolerated, suggesting that the propolis-based mouthwashes can be considered as safe and viable alternatives to CHX.

Definitive conclusions can only be drawn from high quality studies. For this purpose, we critically analyzed the included studies using the Cochrane Collaboration’s tool for assessing risk of bias. The results showed that most of the included studies were of low quality as reflected by the high risk of bias in these studies, making it difficult to draw a robust conclusion. Another concerning issue is the obvious methodological discrepancies in reporting and measuring the outcomes across the included studies. This in fact precluded us from pooling the data and performing meta-analysis.

Adequate follow-up period is central for evaluating the clinical efficacy of any mouthwash [26]. In the present review, the follow-up period was highly variable among the included studies: 5 days in three studies, 14 days in two studies, 21 days in one study, 28 days in 2 studies and 6 weeks days in one study (Table 1). Apart from being inconsistent, the overall follow-up time adopted by all included studies was rather short, considering the 6 month- evaluation period recommended by the American Dental Association (ADA) [26]. Obviously, this is a remarkable limitation of the present study, which further weakens the obtained evidence.

Certainly, the results of the present systematic review support the clinical efficacy of propolis-based mouthwashes for controlling plaque and gingivitis. However, there are many limitations that should be acknowledged. The main limitation is the small sample sizes along with low quality of some of the included studies. Another important limitation is the substantial heterogeneity among the included studies with respect to outcomes measurements, duration of evaluation, dose and formulations of the propolis-based mouthwashes, and gender and age of the participants. Furthermore, the present systematic review included only studies published in English and searching only three databases for the relevant studies and thus we may have missed some potential studies.

## Conclusion

The present study suggests that propolis-based mouthwashes may have good clinical efficacy in reducing plaque and gingival inflammation. However, due to the marked methodological variability across the included studies as well as the high risk of bias in some of the included studies, further well-designed clinical trials with adequate follow-up period and standardized methodologies are highly recommended to discern the clinical efficacy of propolis-based mouthwashes before advising to use them in clinical practice.

## Supplementary information

**Additional file 1: Table 1.** Excluded studies and reasons for exclusion.

**Additional file 2: Table 2.** Means and standard deviations of PI and GI of the included studies.

## Data Availability

The datasets generated and/or analyzed during the current study are available as supplementary tables 1 and 2.

## Abbreviations

CHX: Chlorhexidine
PRISMA: Preferred Reporting Items for Systematic Reviews and Meta-Analyses
PICO: Patient, Intervention, Comparison, and Outcome
RCT: Randomized Controlled Trail
MeSH: Medical Subject Headings
