# Supplementary material for: Efficacy of propolis-based mouthwashes on dental plaque and gingival inflammation: a systematic review
Source: BMC Oral Health. 2020 Jul 10;20:198. doi: 10.1186/s12903-020-01185-5 (PMC7350560; doi:10.1186/s12903-020-01185-5)
Supplement: Supplementary file 1 — Additional file 1: Table 1. Excluded studies and reasons for exclusion. [file 12903_2020_1185_MOESM1_ESM.docx]

**Table 1. Excluded studies and reasons for exclusion**

| Study | Title | Reasons for exclusion |
| --- | --- | --- |
| Darwita et al.  2019[[1](#_ENREF_1)] | Association of propolis fluoride with arrested dentinal caries and dental plaque levels | Topical gels |
| Peycheva et al.  2019[[2](#_ENREF_2)] | Changes in the cytokine levels in adolescents with gingivitis after treatment with propolis | No CHX control group |
| Pereira et al. 2011[[3](#_ENREF_3)] | Clinical Evidence of the Efficacy of a Mouthwash Containing Propolis for the Control of Plaque and Gingivitis: A Phase II Study | No CHX control group |
| [Kumar](https://www.ncbi.nlm.nih.gov/pubmed/?term=Kumar%20A%5BAuthor%5D&cauthor=true&cauthor_uid=26501001) et al. 2015[[4](#_ENREF_4)] | Comparison of Plaque Inhibiting Efficacies of Aloe Vera and Propolis Tooth Gels: A Randomized PCR Study | Gel form& No Chlorhexidine group |
| Jaime et al. 2002[[5](#_ENREF_5)] | Effect of a Mouthrinse Containing Selected Propolis on 3-Day Dental Plaque Accumulation and Polysaccharide Formation. | No CHX control group |
| Pienidhek et al. 2013[[6](#_ENREF_6)] | Influence of Propolis on Hygiene, Gingival Condition, and  Oral Microflora in Patients with Cleft Lip and Palate Treated  With Fixed Orthodontic Appliances | Toothpaste with propolis |
| Tanasiewicz et al. 2012[[7](#_ENREF_7)] | Influence of Hygienic PreparationsWith a 3% Content of Ethanol Extract of Brazilian Propolis on the State of the Oral Cavity | Toothpaste+gel+ |
| Wiatrak et al. 2017[[8](#_ENREF_8)] | Oral Health of Patients Treated with Acrylic Partial Dentures  Using a Toothpaste Containing Bee Product | Toothpaste |
| Samad et al. 2017[[9](#_ENREF_9)] | Propolis Trigona sp. Mouthwash Efectiveness in Lowering Anaerobic Gram-Negative Bacteria Colonies | No CHX control group |
| El-Sharkawy et al. 2016[[10](#_ENREF_10)] | Propolis Improves Periodontal Status and Glycemic Control in subjectswith Type 2 Diabetes Mellitus and Chronic Periodontitis:A Randomized Clinical Trial | Capsules+ No CHX comparison |
| Skaba et al. 2013[[11](#_ENREF_11)] | Influence of the Toothpaste with Brazilian Ethanol Extract Propolis on the Oral Cavity Health | Toothpaste |
| Vervellea et al. 2009[[12](#_ENREF_12)] | Mouthwash solutions with microencapsuled natural extracts:Efficiency for dental plaque and gingivitis | Other language |
| Pundir et al. **2019**[[13](#_ENREF_13)] | One‑stage Full Mouth Disinfection Using 20% PropolisHydroalcoholic Solution: A Clinico‑microbiologic Study | No CHX control group |
| Peychevaa et al. 2019[[14](#_ENREF_14)] | Effect of Bulgarian propolis on the oral microflora in adolescents with plaque-induced gingivitis | No CHX control group |
| [Atwa](https://www.ncbi.nlm.nih.gov/pubmed/?term=Atwa%20AD%5BAuthor%5D&cauthor=true&cauthor_uid=25057231)  et al. 2014[[15](#_ENREF_15)] | Effect of honey in preventing gingivitis and dental caries in patients undergoing orthodontic treatment | No CHX control group |
| [Malić](https://www.ncbi.nlm.nih.gov/pubmed/?term=Mali%C4%87%20M%5BAuthor%5D&cauthor=true&cauthor_uid=3870412)  et al. 1985[[16](#_ENREF_16)] | Effect of Propolis-gel in the treatment of gingival inflammation | Article in Croatian |
| Suriamah et al. 2019[[17](#_ENREF_17)] | Effectiveness of toothpaste containing propolis, tea tree oil, and sodium monofluorophosphate against plaque and gingivitis. | No CHX comparison |
| [Pieniążek](https://www.hindawi.com/19348472/) et al. 2016[[18](#_ENREF_18)] | Effects of Brazilian Propolis on Dental Plaque and Gingiva in Patients with Oral Cleft Malformation Treated with Multibracket and Removable Appliances: A Comparative Study | No CHX comparison |
| Sparabombe et al. 2019[[19](#_ENREF_19)] | Efficacy of an All-Natural Polyherbal Mouthwash in Patients With Periodontitis: A Single-Blind Randomized Controlled Trial | No CHX comparison |
| Taib et al. 2018[[20](#_ENREF_20)] | Evaluation of the complementary effect of tualang honey to non-surgical periodontal therapy: Clinical application | No CHX control group |
| Sanghani et al.[[21](#_ENREF_21)] | Health from the Hive: Propolis as an Adjuvant in the Treatment of Chronic Periodontitis - A Clinicomicrobiologic Study | Periodontitis  No Chlorhexidine |
| Coutinho et al. 2012[[22](#_ENREF_22)] | Honeybee propolis extract in periodontal treatment: a clinical and microbiological study of propolis in periodontal treatment | No CHX controls |
| Piekarz et al 2017[[23](#_ENREF_23)] | The Influence of Toothpaste Containing Australian Melaleuca alternifolia Oil and Ethanolic Extract of Polish Propolis on Oral Hygiene and Microbiome in Patients Requiring Conservative Procedures. | Toothpaste |
| Niedzielska et al 2016[[24](#_ENREF_24)] | The Influence of Ethanolic Extract of Brazilian Green Propolis Gel on Hygiene and Oral Microbiota in Patients after Mandible Fractures. | Topical gels |
| Baumgartner et al 2009[[25](#_ENREF_25)] | The Impact of the Stone Age Diet on Gingival Conditions in the Absence of Oral Hygiene | Irrelevant |
| Bretz et al. 2014[[26](#_ENREF_26)] | The effectiveness of propolis on gingivitis: A randomized controlled trial | No CHX control group |
| M.alibasyah et al. 2018[[27](#_ENREF_27)] | The comparison between dental plaque score before and after gargling with tongra original honey 5% solution (Study of Student in Dentistry of Syiah Kuala University) | No CHX control group |
| Nuray Ercan et al. 2015[[28](#_ENREF_28)] | The comparative effect of propolis in two different vehicles; mouthwash and chewing-gum on plaque accumulation and gingival inflammation | No CHX control group |
| Tadeusz Morawiec et al. 2013[[29](#_ENREF_29)] | The biological activity of propolis-containing toothpaste on oral health environment in patients who underwent implant-supported prosthodontic rehabilitation. | Toothpaste |
| Nagesh Bhat et al. 2015[[30](#_ENREF_30)] | The antiplaque efficacy of propolis-based herbal toothpaste: A crossover clinical study. | Toothpaste |
| Freires et al. 2018[[31](#_ENREF_31)] | The alveolar bone protective effects of natural products: A systematic review | Review article |
| El-sharkawy et al. 2017[[32](#_ENREF_32)] | Systemic Propolis (Adjuvant to Nonsurgical Periodontal Treatment) May aid in Glycemic Control and Periodontal Health in Type 2 Diabetes of Long Duration | Systemic propolis, |
| Dennia perez de Andrade et al. 2017[[33](#_ENREF_33)] | Subgingival Irrigation with a Solution of 20% Propolis Extract as an Adjunct to Non-Surgical Periodontal Treatment: A Preliminary Study. | No CHX control group |
|  | CHX: chlorhexidine |  |

**References**

1. Darwita RR, Setiawati F, Andiani SG: **Association of Propolis Fluoride with Arrested Dentinal Caries and Dental Plaque Levels**. *Journal of International Dental and Medical Research* 2019, **12**(2):622-627.

2. Peycheva S, Apostolova E, Peychev Z, Gardjeva P, Slavov A, Murdjeva M: **CHANGES IN THE CYTOKINE LEVELS IN ADOLESCENTS WITH GINGIVITIS AFTER TREATMENT WITH PROPOLIS**. *FARMACIA* 2019, **67**(2):360-366.

3. Pereira EMR, da Silva JLDC, Silva FF, De Luca MP, Lorentz TCM, Santos VR: **Clinical evidence of the efficacy of a mouthwash containing propolis for the control of plaque and gingivitis: a phase II study**. *Evidence-Based Complementary and Alternative Medicine* 2011, **2011**.

4. Kumar A, Sunkara MS, Pantareddy I, Sudhakar S: **Comparison of plaque inhibiting efficacies of Aloe vera and propolis tooth gels: A randomized PCR study**. *Journal of clinical and diagnostic research: JCDR* 2015, **9**(9):ZC01.

5. Koo H, Cury JA, Rosalen PL, Ambrosano GM, Ikegaki M, Park YK: **Effect of a mouthrinse containing selected propolis on 3-day dental plaque accumulation and polysaccharide formation**. *Caries Research* 2002, **36**(6):445-448.

6. Machorowska-Pieniążek A, Morawiec T, Mertas A, Tanasiewicz M, Dziedzic A, Król W: **Influence of propolis on hygiene, gingival condition, and oral microflora in patients with cleft lip and palate treated with fixed orthodontic appliances**. *Evidence-Based Complementary and Alternative Medicine* 2013, **2013**.

7. Tanasiewicz M, Skucha-Nowak M, Dawiec M, Król W, Skaba D, Twardawa H: **Influence of hygienic preparations with a 3% content of ethanol extract of Brazilian propolis on the state of the oral cavity**. *Advances in Clinical and Experimental Medicine* 2012, **21**(1):81-92.

8. Wiatrak K, Morawiec T, Rój R, Mertas A, Machorowska-Pieniążek A, Kownacki P, Tanasiewicz M, Skucha-Nowak M, Baron S, Piekarz T: **Oral health of patients treated with acrylic partial dentures using a toothpaste containing bee product**. *Evidence-Based Complementary and Alternative Medicine* 2017, **2017**.

9. Samad R: **Propolis Trigona sp. Mouthwash Efectiveness in Lowering Anaerobic Gram-Negative Bacteria Colonies**. 2017.

10. El‐Sharkawy HM, Anees MM, Van Dyke TE: **Propolis improves periodontal status and glycemic control in patients with type 2 diabetes mellitus and chronic periodontitis: a randomized clinical trial**. *Journal of periodontology* 2016, **87**(12):1418-1426.

11. Skaba D, Morawiec T, Tanasiewicz M, Mertas A, Bobela E, Szliszka E, Skucha-Nowak M, Dawiec M, Yamamoto R, Ishiai S: **Influence of the toothpaste with brazilian ethanol extract propolis on the oral cavity health**. *Evidence-Based Complementary and Alternative Medicine* 2013, **2013**.

12. Vervelle A, Mouhyi J, Del MC, Hippolyte M, Sammartino G, Dohan DE: **Mouthwash solutions with microencapsuled natural extracts: Efficiency for dental plaque and gingivitis**. *Revue de stomatologie et de chirurgie maxillo-faciale* 2010, **111**(3):148-151.

13. Pundir AJ, Vishwanath A, Pundir S, Swati M, Banchhor S, Jabee S: **One-stage full mouth disinfection using 20% propolis hydroalcoholic solution: A clinico-microbiologic study**. *Contemporary clinical dentistry* 2017, **8**(3):416.

14. Peycheva S, Apostolova E, Gardjeva P, Peychev Z, Kokova V, Angelov A, Slavov A, Murdjeva M: **Effect of Bulgarian propolis on the oral microflora in adolescents with plaque-induced gingivitis**. *Revista Brasileira de Farmacognosia* 2019, **29**(3):271-277.

15. Atwa A-DA, AbuShahba RY, Mostafa M, Hashem MI: **Effect of honey in preventing gingivitis and dental caries in patients undergoing orthodontic treatment**. *The Saudi dental journal* 2014, **26**(3):108-114.

16. Malić M, Konjhodzić H, Filipović M: **Effect of Propolis-gel in the treatment of gingival inflammation**. *Stomatoloski vjesnik Stomatological review* 1985, **14**(3-4):107.

17. Suriamah N, Lessang R, Kemal Y: **Effectiveness of toothpaste containing propolis, tea tree oil, and sodium monofluorophosphate against plaque and gingivitis**. *International Journal of Applied Pharmaceutics* 2019, **11**:114-116.

18. Machorowska-Pieniążek A, Skucha-Nowak M, Mertas A, Tanasiewicz M, Niedzielska I, Morawiec T, Baron S: **Effects of brazilian propolis on dental plaque and gingiva in patients with oral cleft malformation treated with multibracket and removable appliances: A comparative study**. *Evidence-Based Complementary and Alternative Medicine* 2016, **2016**.

19. Sparabombe S, Monterubbianesi R, Tosco V, Orilisi G, Hosein A, Ferrante L, Putignano A, Orsini G: **Efficacy of an all-natural polyherbal mouthwash in patients with periodontitis: a single-blind randomized controlled trial**. *Frontiers in physiology* 2019, **10**:632.

20. Taib H, Kamarulzaman N, Min HJ, Berahim Z, Zainuddin SLA: **Evaluation of the Complementary Effect of Tualang Honey to Non-Surgical Periodontal Therapy: Clinical Application**. *Journal of International Dental and Medical Research* 2018, **11**(2):650-655.

21. Sanghani NN, Shivaprasad B, Savita S: **Health from the hive: propolis as an adjuvant in the treatment of chronic periodontitis-a clinicomicrobiologic study**. *Journal of clinical and diagnostic research: JCDR* 2014, **8**(9):ZC41.

22. Coutinho A: **Honeybee propolis extract in periodontal treatment: A clinical and microbiological study of propolis in periodontal treatment**. *Indian Journal of Dental Research* 2012, **23**(2):294.

23. Piekarz T, Mertas A, Wiatrak K, Rój R, Kownacki P, Śmieszek-Wilczewska J, Kopczyńska E, Wrzoł M, Cisowska M, Szliszka E: **The influence of toothpaste containing Australian Melaleuca alternifolia oil and ethanolic extract of polish propolis on oral hygiene and microbiome in patients requiring conservative procedures**. *Molecules* 2017, **22**(11):1957.

24. Niedzielska I, Puszczewicz Z, Mertas A, Niedzielski D, Różanowski B, Baron S, Konopka T, Machorowska-Pieniążek A, Skucha-Nowak M, Tanasiewicz M: **The influence of ethanolic extract of Brazilian green propolis gel on hygiene and oral microbiota in patients after mandible fractures**. *BioMed research international* 2016, **2016**.

25. Baumgartner S, Imfeld T, Schicht O, Rath C, Persson RE, Persson GR: **The impact of the stone age diet on gingival conditions in the absence of oral hygiene**. *Journal of periodontology* 2009, **80**(5):759-768.

26. Bretz WA, Paulino N, Nör JE, Moreira A: **The effectiveness of propolis on gingivitis: a randomized controlled trial**. *The Journal of Alternative and Complementary Medicine* 2014, **20**(12):943-948.

27. Alibasyah ZM, Saputri D, Alviana V: **The Comparison Between Dental Plaque Score Before and After Gargling with Tongra Original Honey 5% Solution (Study of Student in Dentistry of Syiah Kuala University)**. *Biomedical and Pharmacology Journal* 2018, **11**(1):381-385.

28. Ercan N, Erdemir EO, Ozkan SY, Hendek MK: **The comparative effect of propolis in two different vehicles; mouthwash and chewing-gum on plaque accumulation and gingival inflammation**. *European journal of dentistry* 2015, **9**(02):272-276.

29. Morawiec T, Dziedzic A, Niedzielska I, Mertas A, Tanasiewicz M, Skaba D, Kasperski J, Machorowska-Pieniążek A, Kucharzewski M, Szaniawska K: **The biological activity of propolis-containing toothpaste on oral health environment in patients who underwent implant-supported prosthodontic rehabilitation**. *Evidence-based complementary and alternative medicine* 2013, **2013**.

30. Bhat N, Bapat S, Asawa K, Tak M, Chaturvedi P, Gupta VV, George PP: **The antiplaque efficacy of propolis-based herbal toothpaste: A crossover clinical study**. *Journal of natural science, biology, and medicine* 2015, **6**(2):364.

31. Freires IA, Santaella GM, Sardi JdCO, Rosalen PL: **The alveolar bone protective effects of natural products: A systematic review**. *Archives of oral biology* 2018, **87**:196-203.

32. Borgnakke WS: **Systemic Propolis (Adjuvant to Nonsurgical Periodontal Treatment) May aid in Glycemic Control and Periodontal Health in Type 2 Diabetes of Long Duration**. *Journal of Evidence Based Dental Practice* 2017, **17**(2):132-134.

33. de Andrade DP, Carvalho ICS, Godoi BH, Rosa LCL, Barreto LMRC, Pallos D: **Subgingival Irrigation with a Solution of 20% Propolis Extract as an Adjunct to Non-Surgical Periodontal Treatment: A Preliminary Study**. *Journal of the International Academy of Periodontology* 2017, **19**(4):145-151.
