# Supplementary material for: Efficacy of propolis-based mouthwashes on dental plaque and gingival inflammation: a systematic review
Source: BMC Oral Health. 2020 Jul 10;20:198. doi: 10.1186/s12903-020-01185-5 (PMC7350560; doi:10.1186/s12903-020-01185-5)
Supplement: Supplementary file 2 — Additional file 2: Table 2. Means and standard deviations of PI and GI of the included studies. [file 12903_2020_1185_MOESM2_ESM.docx]

**Table 2. Means and standard deviations of PI and GI of the included studies**

| **Author (year)** | **Propolis group** | | | **Chlorhexidine group** | |  |
| --- | --- | --- | --- | --- | --- | --- |
|  |  | baseline | After  treatment | baseline | After  treatment | P-value |
| Murray MC et al 1997 | PI | 0.44(0.26) | 1.02(0.24) | 0.44(0.32) | 0.51(0.28) | >0.001 |
| Santiago  2018 | PHP | 2.6(2.8) | ^2.0(2.2)^ | 3.2(3.4) | 2.0(2.2)^*^  *^SEM^ | NA |
| Porwal et al  2018 | PI | 3.34(0.25) | 0.94(0.57) | 3.26(0.53) | 0.82(0.45) | 0.721 |
|  | MGI | 3.16(0.34) | 0.28(0.25) | 3.04(0.23) | 0.54(0.35) | 0.225 |
| Krishna et al  2019 | PI | 1.95(0.07) | 1.47(0.21) | 1.94( 0.08) | 1.61(0.16) | 0.04 |
|  | GI | 1.86(0.19) | 1.42(0.2) | 1.86(0.19) | 1.53(0.14) | 0.21 |
| Dodwad et al.  2011 | PI | 1.26 | 2.12 | 1.14 | 1.32 | 0.5392 |
|  | GI | 0.86 | 0.92 | 0.94 | 1.02 | 0.0048 |
| Savita  et al  2018 | PI | 1.25 ± 0.09 | 1.12 ± 0.04 | 1.38 ± 0.14 | 1.26 ± 0.13 | 0.001 |
|  | GI | 1.18 ± 0.03 | 1.10 ± 0.03 | 1.30 ±0.11 | 1.12 ± 0.098 | 0.829 |
| Adullah et al  2003 | PI | 1.1 | 0.38 | 0.59 | 0.37 | >0.05 |
| Dehghani et al  2019 | PI | 1.4(0.6) | 0.7(0.5) | 1.5(0.6) | 0.7(0.5) | 0.746 |
|  | GI | 1.7(0.3) | 1.3(0.6) | 1.8(0.5) | 1.2(0.5) | 0.946 |
|  | CPI | 1(0.5) | 0.7(0.4) | 0.9(0.4) | 0.6(0.5) | 0.771 |
| ANAUATE-NETTO et al  2014 | PBS | 1.0(0.5) | 0.5(0.5) | 1.1(0.5) | 0.9(0.6) | >0.05 |
| PI: plaque index; GI: gingival index; NA: not available; ^**^the mean is missing; the authors only reported the range of means. | | | | | |  |
